# Critical role of endogenous histamine in promoting end-organ tissue injury in sepsis

**DOI:** 10.1186/s40635-016-0109-y

**Published:** 2016-11-08

**Authors:** Mizuki Hattori, Mitsuaki Yamazaki, Wakana Ohashi, Satoshi Tanaka, Kohshi Hattori, Kenichiro Todoroki, Toshio Fujimori, Hiroshi Ohtsu, Naoyuki Matsuda, Yuichi Hattori

**Affiliations:** 1 Department of Molecular and Medical Pharmacology, Graduate School of Medicine and Pharmaceutical Sciences, University of Toyama, Toyama, Japan; 2 Department of Anesthesiology, Graduate School of Medicine and Pharmaceutical Sciences, University of Toyama, Toyama, Japan; 3 Department of Immunobiology, Division of Pharmaceutical Sciences, Okayama University Graduate School of Medicine, Dentistry, and Pharmaceutical Sciences, Okayama, Japan; 4 Department of Anesthesiology and Pain Relief Center, The University of Tokyo Hospital, Tokyo, Japan; 5 Department of Analytical and Bio-Analytical Chemistry, School of Pharmaceutical Sciences, University of Shizuoka, Shizuoka, Japan; 6 Department of Applied Quantum Medical Engineering, School of Engineering, Tohoku University, Sendai, Japan; 7 Department of Emergency and Critical Care Medicine, Nagoya University Graduate School of Medicine, Nagoya, Japan

**Keywords:** Sepsis, Histamine, Histidine decarboxylase, Organ injury

## Abstract

**Background:**

Histamine assumes an important role as a major mediator in various pathologic disorders associated with inflammation and immune reactions. However, the involvement of histamine in the pathological conditions and symptoms of sepsis remains entirely unknown. In this study, we establish that histamine is identified as a contributory mediator to promoting the development of organ injury in sepsis.

**Methods:**

Histidine decarboxylase (HDC) gene knockout (HDC^−/−^) mice, histamine H_1_-/H_2_-receptor gene-double knockout (H_1_R^−/−^/H_2_R^−/−^) mice, and their littermate wild-type (WT) C57BL/6J mice underwent cecal ligation and puncture (CLP) or sham operation. Some WT mice were injected intraperitoneally with *d*-chlorpheniramine and famotidine 60 min before CLP to block H_1_- and H_2_-receptors, respectively.

**Results:**

In mice rendered septic by CLP, tissue histamine levels were elevated in association with increased HDC expression. Sepsis-induced abnormal cytokine production and multiple organ injury (lung, liver, and kidney) were significantly less pronounced in HDC^−/−^ mice as compared with WT controls, and HDC deficiency had improved survival in sepsis. This benefit corresponded with a significant reduction in activation levels of the nuclear factor (NF)-κB signaling pathway. H_1_R^−/−^/H_2_R^−/−^ mice apparently behaved similar to HDC knockout mice in reducing sepsis-related pathological changes. Pharmacological interventions with H_1_- and H_2_-receptor antagonists indicated that both H_1_- and H_2_-receptors were involved in septic lung and liver injury, whereas only H_2_-receptors contributed to septic kidney injury.

**Conclusions:**

In the setting of sepsis, histamine, through activation of H_1_- and H_2_-receptors, serves as an aggravating mediator to contribute to the development of sepsis-driven major end-organ failure.

**Electronic supplementary material:**

The online version of this article (doi:10.1186/s40635-016-0109-y) contains supplementary material, which is available to authorized users.

## Background

Sepsis is a common and potentially life-threatening medical condition in populations in intensive care units (ICU). Despite advances in overall care of critically ill patients, sepsis remains the primary cause of death from microbial infections [1, 2]. The development of a failure of one or more organs, including the lung, kidney, and liver, poses a major threat to the survival of patients with sepsis. In accordance with the importance of more timely management of patients with sepsis or at risk of developing sepsis, sepsis is now defined as life-threatening organ dysfunction due to a dysregulated host response to infection [3]. The pathogenesis of sepsis-induced organ failure has been extensively gleaned from animal models and human studies [4–6], but the mechanisms underlying the pathophysiologic processes that both initiate and promulgate organ dysfunction in sepsis have not been fully elucidated. A greater understanding of the mechanisms that underlie the development of organ dysfunction in sepsis may enable us to develop therapies targeted at preventing or limiting molecular events associated with the progress of fatal organ failure and, hence, leading to improved outcomes.

In a prospective, controlled, clinical study, elevated plasma histamine levels have been shown to be causally associated with sepsis [7]. In our previous studies, the sustained elevation of plasma histamine has been shown to be associated with the time-dependent increase in expression of histidine decarboxylase (HDC), which is the catabolic enzyme of histamine synthesis, in the animals with lipopolysaccharide (LPS)- and cecal ligation and puncture (CLP)-induced sepsis [8–10]. Furthermore, endotoxemia may cause superinduction of H_1_- and H_2_-receptors in cardiovascular and pulmonary tissues [8, 9, 11]. Since histamine mediates a wide range of cellular responses, including allergic and inflammatory reactions, gastric acid secretion, vascular tone and permeability, and neurotransmission in the central nervous system [12], the histamine biological responsiveness may be of special importance in certain pathological aspects suggestive of histamine release. It would be thus allowable to assume that histamine may play a contributory role in the development of major organ dysfunction and failure associated with sepsis.

In the present study, we examined whether genetic and pharmacological interventions of histamine can provide a change in systemic inflammation and organ injury in mice with CLP-induced polymicrobial sepsis in order to explore the role of histamine in the pathophysiology of the septic syndrome. CLP-induced sepsis is an animal model that has high relevance to humans because it reproduces many hallmarks of sepsis that occur in patients [13]. We applied HDC gene knockout (HDC^−/−^) mice [14], lacking histamine, to investigate the effect of histamine deficiency on the pathophysiology of CLP-induced sepsis. Along with HDC^−/−^ mice, we also used histamine H_1_-/H_2_-receptor gene-double knockout (H_1_R^−/−^/H_2_R^−/−^) mice generated by crossbreeding of H_1_-receptor null mice and H_2_-receptor null mice. Finally, we tested changes in the pathophysiological features of CLP-induced sepsis by pharmacological antagonism of H_1_- and H_2_-receptors.

## Methods

### Generation of HDC^−/−^ mice and H_1_R^−/−^/H_2_R^−/−^ mice

HDC^−/−^ mice were generated according to previously described procedures [14]. H_1_-receptor gene deficient mice and H_2_-receptor gene deficient mice were a gift from Prof. Kazuhiko Yanai, Tohoku University [15, 16], and the progeny of the colony was maintained. Serially breeding of these two strains generated the double-knockout line (H_1_R^−/−^/H_2_R^−/−^). Genotyping of the resultant mice was determined by PCR analysis of DNA extracted from tail samples. HDC^−/−^ mice and H_1_R^−/−^/H_2_R^−/−^ mice were of a genetic background of a C57BL/6 J strain, and their littermates were used as wild-type (WT) controls. Mice were housed under specific-pathogen-free conditions.

### Animal model of sepsis

All animal studies were conducted in accordance with the National Institute of Health Guidelines on the use of laboratory animal and with approval of the Care and Use Committee of the University of Toyama. The surgical procedure to generate CLP-induced sepsis was performed as described elsewhere [17–19]. In brief, male mice, 8–10 weeks old, were anesthetized with 3–4% sevoflurane, and a middle abdominal incision was made. The cecum was mobilized, ligated, and punctured twice with a 21-gauge needle, allowing exposure of faces, the bowel was repositioned, and the abdomen was closed with sterile suture. Sham-operated control underwent the same procedure except for ligation and puncture of the cecum. Some WT mice were injected intraperitoneally with a single dose of *d*-chlorpheniramine (10 mg/kg) and famotidine (20 mg/kg) 60 min before CLP to block H_1_- and H_2_-receptors, respectively. A noninvasive computerized tail-cuff system was used for measuring blood pressure and heart rate in mice [17, 20].

### Measurement of histamine

The amount of histamine was determined by the fluorometrical method with *o*-phthalaldehyde [21]. The tissues were homogenized in 4–5 volumes of PBS containing 2 M NaCl, lysed using 0.5% Triton X-100, and centrifuged at 12,000×*g* for 30 min at 4 °C in order to obtain the soluble fraction for histamine assay.

### RNA extraction and quantitative real-time PCR

Total RNA was isolated from tissues with Sepazol-RNA I Super G (Nacalai Tesque, Kyoto, Japan). PrimeScript RT Master Mix (Takara Bio, Ohtsu, Japan) or ReverTra Ace qPCR RT Master Mix (Toyobo, Osaka, Japan) was used for the reverse transcription reaction, and real-time PCR analyses were performed using SYBR Premix Ex Taq II (Tli RNaseH Plus), ROX plus (Takara Bio). Values were normalized to glyceraldehyde-3-phosphate dehydrogenase (GAPDH) according to the manufacturer’s protocol (MX3000P real-time PCR system; Agilent Technologies Inc., Santa Clara, CA, USA).

### Serum analysis

Blood was collected in serum gel tubes (Sarsted, Nümbrecht, Germany), and serum was obtained and stored at −80 °C. The quantitative determination of aspartate aminotransferase (AST), alanine aminotransferase (ALT), blood urea nitrogen (BUN), and creatinine in serum was made on Hitachi 7180 Biochemistry Automatic Analyzer (Hitachi High-Technologies, Tokyo, Japan). Interleukin (IL)-1β, IL-6, tumor necrosis factor (TNF)-α, and monocyte chemotactic protein (MCP)-1 were measured by the use of a commercially available enzyme-linked immunosorbent assay (ELISA) kit (R&D Systems, Minneapolis, MN, USA), according to the manufacturer’s instructions. The plate was read on a microplate reader (Nippon-InterMed, Tokyo, Japan). Assays were performed in duplicate.

### Lung wet-to-dry weight ratio

Surgically removed lung tissues were blotted dry and weighed to determine the lung wet weight. The lung tissues were then wrapped loosely in aluminum foil, placed in a drying oven overnight, and weighed again for calculation of the wet-to-dry weight ratio [9, 22].

### Histologic examination

Tissues were fixed by immersion in 10 % buffered formaldehyde overnight, embedded in paraffin, and cut into 4-μm-thick sections. After deparaffinization, slides were stained with hematoxylin and eosin by standard methods. All the histological studies were performed in a blinded fashion. A semiquantitative morphometric analysis of lung injury was performed by scoring from 0 to 4 (none, light, moderate, severe, very severe) for the following categories: neutrophil infiltration, pulmonary edema, and disorganization of lung parenchyma and hemorrhage [17]. A total lung injury score was calculated by adding the individual scores in every animal and averaging the total values in each group.

### Immunohistochemistry

Tissue sections (4 μm) were rehydrated, and endogenous peroxidases were quenched with 3% hydrogen peroxide. Slides were then incubated overnight at 4 °C with primary antibodies for myeloperoxidase (MPO; 1:200 dilution; Abcam, Cambridge, MA, USA), or neutrophil gelatinase-associated lipocalin (NGAL; 1:2000; Abcam). All sections were incubated with Histofine® Simple Stain Mouse MAX PO(R) (Nichirei Biosciences, Tokyo, Japan) including the secondary antibody which is reduced to Fab fragment. Sections were developed with 3,3’-diaminobenzidine and counterstained with hematoxylin.

### Immunofluorescence staining

The tissue sections were exposed to the fluorescent antibody Alexa Fluor 546-conjugated anti-mouse IgG (Invitrogen, Carlsbad, CA, USA) after overnight incubation with the primary antibody according to the method in our previous study with minor modification [20]. The nucleus was counterstained with Hoechst 33342 dye (Invitrogen). Immunofluorescence images were observed under an Olympus BX-51 fluorescence microscope (Olympus, Tokyo, Japan) and processed using Adobe Photoshop CC software (Adobe, San Jose, CA, USA).

### Western blot analysis

After being removed and rinsed in sterilized PBS on ice, tissues were homogenized and then centrifuged at 18,000×*g* for 10 min at 4 °C, and the resulting supernatants were collected. When required, nuclear protein extracts from lungs were obtained with a commercially available nuclear extraction kit (Sigma-Aldrich, St. Louis, MO, USA), as described in the manufacturer’s manual. The proteins in the supernatant were measured using BCA Protein Assay Kit (Thermo Fisher Scientific, Rockford, IL, USA). Immunoblotting was performed as described in our previous reports [19, 23]. Samples (30–50 μg of protein) were electrophoresed on 10 or 14 % SDS-PAGEs and transferred to PVDF membrane. For primary antibody incubation (overnight at 4 °C), rabbit polyclonal or monoclonal antibodies were used against NGAL (1:1,000; Abcam), IκBα (1:1,000; Cell Signaling, Danvers, MA, USA), and phospho-IκBα (Ser-32) (1:1,000; Cell Signaling), whereas a mouse monoclonal antibody was used against nuclear factor (NF)-κB (1:200; Santa Cruz Biotechnology, Santa Cruz, CA, USA), β-actin (1:5,000; Wako Pure Chemical, Osaka, Japan), and GAPDH (1:5,000; Wako Pure Chemical) and a goat polyclonal antibody against lamin B (1:200; Santa Cruz Biotechnology). Primary antibody detection was performed with horseradish peroxidase-conjugated secondary antibodies. Binding of the antibody was detected by an ImmunoStar Zeta (Wako Pure Chemical), and levels of protein expression were quantitated by a luminoimage LAS-4000 analyzer (Fuji Film, Tokyo, Japan).

### Statistical analysis

Values are expressed as means ± SEM. Statistical assessment of the data was made by Student’s unpaired *t* test or ANOVA followed by Tukey’s multiple comparison test using Prism software (ver. 7; GraphPad Software, San Diego, CA, USA). Differences at *p* < 0.05 were considered statistically significant.

## Results

### Changes in tissue histamine concentrations, HDC expression, and histamine receptor expression after sepsis induction

**Fig. 1:**
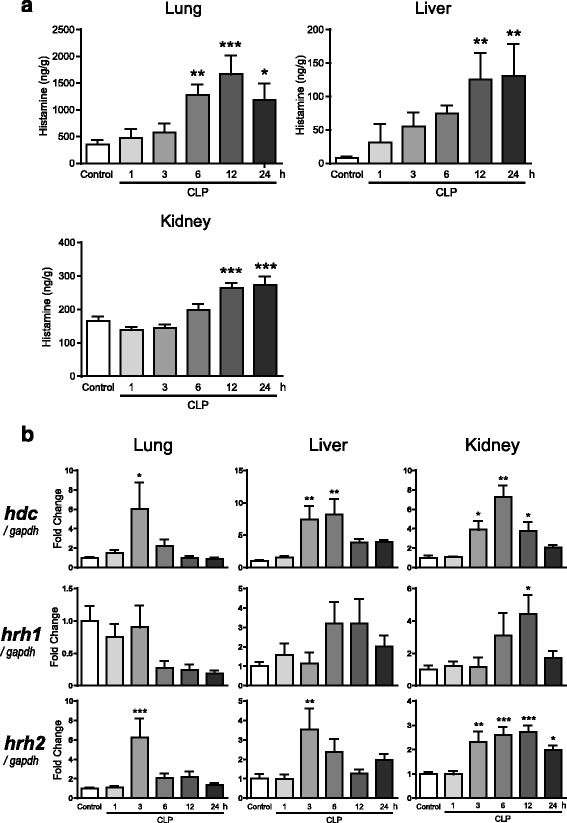
Changes in histamine synthesis and histamine receptor expression in lung, liver, and kidney tissues from mice after CLP-induced sepsis. **a** Tissue histamine concentrations after CLP (*n* = 8/group). **b** Tissue mRNA levels of HDC, histamine H_1_- and H_2_-receptors after CLP (*n* = 6/group). The mRNA levels were quantified by real-time PCR. The values were expressed as a fold increase above control normalized GAPDH. All values are provided as means ± SEM. *P < 0.05, **P < 0.01, and ***P < 0.001 vs. control

We initially ascertained whether tissue histamine synthesis is altered in WT mice after sepsis induction by CLP. As demonstrated in our previous report [10], CLP-induced polymicrobial sepsis resulted in an increase in plasma concentrations of histamine in mice (Additional file 1: Figure S1). Thus, plasma histamine concentrations were significantly (*p* < 0.05) elevated from baseline of 16.1 ± 3.5 ng/mL (*n* = 4) early after CLP, with a peak concentration at 3 h (38.9 ± 3.5 ng/mL, *n* = 4). The basal levels of histamine highly varied between tissues (lung, 349 ± 87 ng/g; liver, 7.9 ± 2.7 ng/g; kidney, 165 ± 14 ng/g, *n* = 13 for each). When sepsis was induced by CLP, however, histamine levels elevated in all tissues in a time-dependent manner (Fig. 1a). In mammalian tissues, histamine is synthesized from L-histidine by HDC. Real-time PCR analysis showed that the transcript levels of HDC were transiently but greatly increased in all tissues after induction of sepsis (Fig. 1b).

Changes in histamine H_1_- and H_2_-receptor mRNA expression in the lung, liver, and kidney tissues of WT mice after sepsis induction were also examined by real-time PCR (Fig. 1b). In lung tissues, no increase in H_1_-receptor mRNA expression was observed after CLP. On the other hand, the mRNA levels were increased more than threefold in liver and kidney tissues at 6–12 h after CLP when compared with those shown in controls. Following induction of sepsis by CLP, a significant increase in H_2_-receptor mRNA expression was transiently detected in lung and liver tissues. In the kidney, CLP-induced sepsis resulted in a sustained, significant increase in the transcript level of H_2_-receptors.

### Sepsis-induced inflammation and organ injury are alleviated in HDC knockout mice

**Fig. 2:**
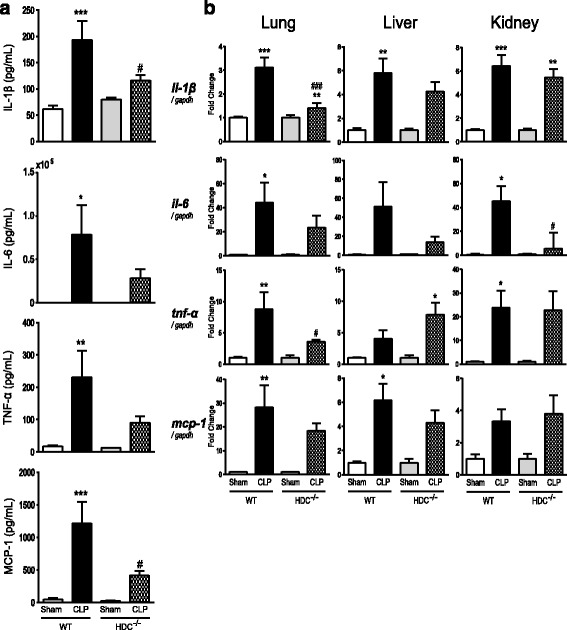
Reduced cytokine levels in HDC^−/−^ mice following CLP-induced sepsis. **a** Blood levels of IL-1β, IL-6, TNF-α, and MCP-1. The blood was collected 18 h after surgery (*n* = 4–9/group), and those cytokine levels were measured by the use of ELISA. **b** Transcription levels of IL-1β, IL-6, TNF-α, and MCP-1 in lung, liver, and kidney tissues. Tissues were harvested 18 h after surgery (*n* = 4–11/group). The mRNA levels were quantified by real-time PCR. The values were expressed as a fold increase above sham-operated control normalized GAPDH. All values are provided as means ± SEM. *P < 0.05, **P < 0.01, and ***P < 0.001 vs. the respective control (18 h after sham operation). ^#^ *P* < 0.05 vs. CLP WT

When blood levels of proinflammatory or chemotactic cytokines were measured using an ELISA, the sham-operated control animals had low levels of the cytokines examined here and no difference was found between WT and HDC^−/−^ mice (Fig. 2a). The animals 18 h after CLP-induced sepsis had marked elevations in IL-1β, IL-6, TNF-α, and MCP-1. Following sepsis induction, however, HDC^−/−^ mice displayed an evidently lower levels of those cytokines compared with WT mice. We also examined changes in mRNA levels of IL-1β, IL-6, TNF-α, and MCP-1 in lung, liver, and kidney tissues using real-time PCR (Fig. 2b). After induction of sepsis, mRNA expression levels of those cytokines greatly increased in all tissues.

**Fig. 3:**
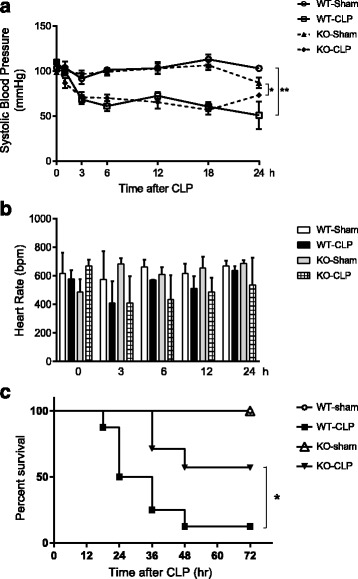
Hypotension, heart rate, and mortality in WT and HDC^−/−^ mice with CLP-induced sepsis. **a** Systolic arterial blood pressure (SBP) after surgery. **b** Heart rate changes after surgery. All values are provided as means ± SEM. *p < 0.05, **p < 0.01; *n* = 3–5 per group. **c** Kaplan-Meier survival curves. Eight mice were used for each group. *p < 0.05 (log rank test)

The animals subjected to CLP showed a sharp fall in systolic blood pressure (Fig. 3a). No significant difference in hypotension was observed between WT and HDC^−/−^ mice after CLP. The CLP-induced sepsis caused a transient decrease in the heart rate in both WT and HDC^−/−^ mice, but the heart rate responses of the two animal groups were not substantially different (Fig. 3b). HDC^−/−^ mice had a survival advantage after CLP as compared with WT controls (Fig. 3c).

**Fig. 4:**
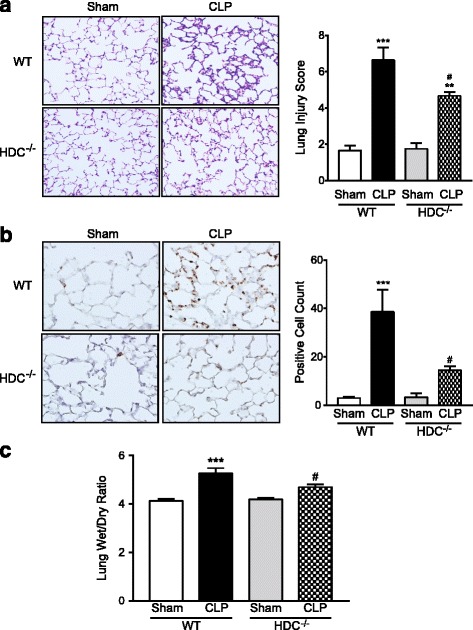
Reduced lung injury in HDC^−/−^ mice following CLP-induced sepsis. Lung tissues were harvested from sham-operated and CLP-induced septic mice 24 h after surgery. **a** Lung sections stained with hematoxylin and eosin. Original magnification, ×200. A bar graph shows semiquantitative analysis of lung tissues by lung injury score, which was performed by scoring from 0–4 as described in Methods. A total lung injury score was calculated by adding the individual scores in every animal and averaging the total values in each group (*n* = 4–10/group). **b** Sections were stained with antibody against MPO followed by peroxidase staining. Original magnification, x200 or x400. A bar graph shows the summary of quantitation of MPO-positive cell counts. The average of MPO-positive cell number in three fields per sample was calculated (*n* = 4–10/group). **c** Wet-to-day ratios of lungs harvested from the animals were determined to assess pulmonary edema (*n* = 6/group). The summarized results are presented as means ± SEM. *P < 0.05, **P < 0.01, and ***P < 0.001 vs. the respective control (24 h after sham operation). ^##^ *P* < 0.01 vs. CLP WT

Histologic examination of hematoxylin and eosin-stained sections of the lungs showed massive infiltration of inflammatory cells, disorganized architecture with irregular alveoli, and intra-alveolar hemorrhage arising from capillary rupture in WT mice 24 h after sepsis induction by CLP (Fig. 4a). In lungs from HDC^−/−^ mice, these histopathological changes were lessened. Semiquantitative assessment using lung injury score revealed that the score was significantly lower in HDC^−/−^ mice than in WT controls. The sepsis-induced increase in lung staining of MPO, an index of neutrophil infiltration, was significantly reduced in HDC^−/−^ mice in comparison with WT controls (Fig. 4b). When the wet-to-dry lung weight ratio was measured for assessment of lung vascular leak, the ratio was significantly increased in WT mice after sepsis induction (Fig. 4c).

**Fig. 5:**
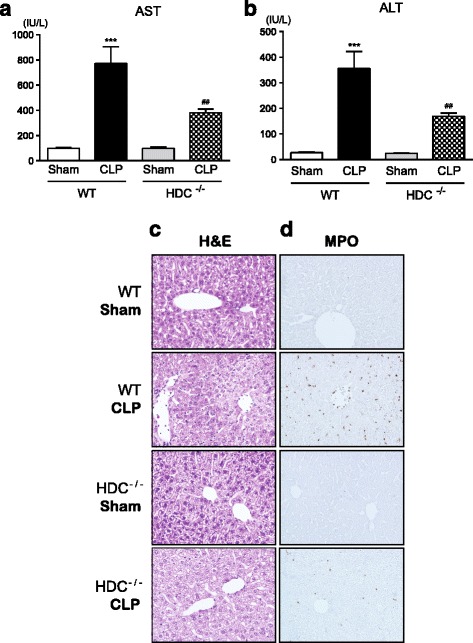
Reduced liver injury in HDC^−/−^ mice following CLP-induced sepsis. **a**, **b** Serum levels of AST and ALT. Blood samples were collected from sham-operated and CLP-induced septic mice 18 h after surgery (*n* = 4–11/group). All values are provided as means ± SEM. ***P < 0.001 vs. the respective control (18 h after sham operation). ^##^ *P* < 0.01 vs. CLP WT. **c**, **d** Representative micrographs liver sections stained with hematoxylin and eosin and anti-MPO antibody followed by peroxidase staining. Original magnification, ×200. Tissues were harvested from sham-operated and CLP-induced septic mice 24 h after surgery. The same results were obtained with two other experiments

Following induction of sepsis by CLP, a marked elevation in serum levels of AST and ALT, a functional readout for liver damage, was observed in WT mice (Fig. 5a). The elevation in these serum aminotransferase levels after sepsis was significantly lowered in HDC^−/−^ mice. When liver injury was assessed using liver specimens stained with hematoxylin and eosin, massive alterations in hepatocytes, including irregular contour of cells and nuclei, cytoplasmic vacuolation, cytoplasmic and nuclear degeneration, and cellular rupture, were found in WT mice after sepsis induction (Fig. 5b). A destruction of the sinusoidal structure of the liver and erythrocyte agglutination were also observed. Such histopathological alterations showing the liver damage after sepsis was less pronounced in HDC^−/−^ mice. Furthermore, the highly increased neutrophilic influx in the liver from septic WT mice was indicated by MPO staining (Fig. 5c). There was much lower MPO expression in liver specimens from HDC^−/−^ mice.

**Fig. 6:**
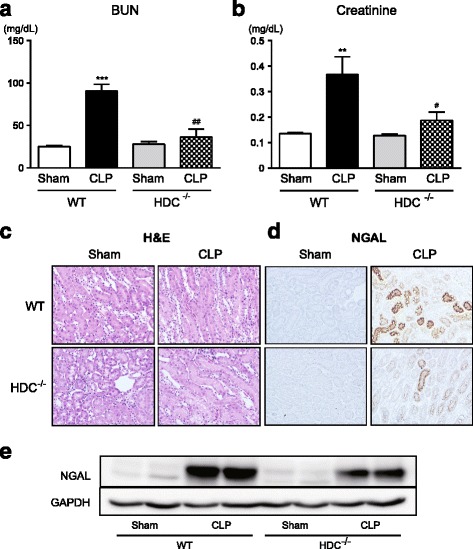
Reduced kidney injury in HDC^−/−^ mice following CLP-induced sepsis. **a**, **b** Serum levels of BUN and creatinine. Blood samples were collected from sham-operated and CLP-induced septic mice 18 h after surgery (*n* = 4–11/group). All values are provided as means ± SEM. ***P < 0.001 vs. the respective control (18 h after sham operation). ^#^ *P* < 0.05 and ^##^ *P* < 0.01 vs. CLP WT. **c**, **d** Representative micrographs liver sections stained with hematoxylin and eosin and anti-NGAL antibody followed by peroxidase staining. Original magnification, ×200. The same results were obtained with two other experiments. **e** Western blot image of NGAL protein expression. GAPDH served as loading control. Shown are representative blots from three independent experiments in which the same results were obtained. Tissues were harvested from sham-operated and CLP-induced septic mice 24 h after surgery

The serum levels of BUN and creatinine, both of which provide a guide to kidney function, were markedly elevated in septic WT mice (Fig. 6a, b). Pathologically elevated serum BUN and creatinine levels were reduced in HDC^−/−^ mice. No apparent histopathological finding was detectable even in WT mice after sepsis induction when the renal tissue sections were stained using hematoxylin and eosin (Fig. 6c). However, we found that septic WT mice displayed the intense staining of NGAL, a biomarker of kidney damage (Fig. 6d). HDC^−/−^ mice following sepsis induction exhibited weaker NGAL staining in kidneys. In line with the findings from immunohistochemical assessment of renal NGAL, Western blot analysis showed that a striking rise in renal expression of NGAL caused by sepsis was more evident in WT as compared with HDC^−/−^ mice (Fig. 6e).

### Sepsis-induced NF-κB activation is reduced in HDC knockout mice

**Fig. 7:**
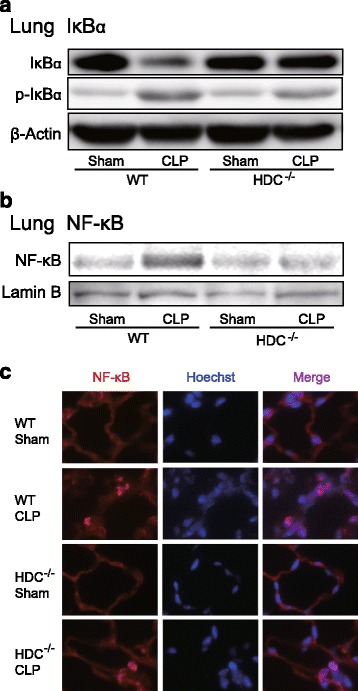
Kinetics of NF-κB activation in lungs of HDC^−/−^ mice following CLP-induced sepsis. Lung tissues were harvested from sham-operated and CLP-induced septic mice 18 h after surgery. **a** Western blot analysis using anti-IκBα antibody and anti-phospho-IκBα antibody. β-Actin served as loading control. **b** Nuclear proteins were extracted, and then NF-κB p65 was detected by Western blot analysis. Lamin B served as a nuclear marker. **c** Immunofluorescent images for NF-κB p65 (*red*) in lung sections. Nuclei were counterstained with Hoechst 33342 dye (*blue*). Original magnification, x400. Shown are representative blots from two independent experiments in which the same results were obtained

We examined whether sepsis-induced activation of the transcription factor NF-κB is altered in HDC^−/−^ mice. Since the activity of NF-κB is primarily regulated by interaction with its inhibitory protein IκBα, phosphorylation and degradation of IκBα in lung tissues after sepsis induction were monitored by Western blot (Fig. 7a). Induction of sepsis resulted in greatly increased phosphorylation and degradation of IκBα in lungs of WT mice. Such changes were diminished in HDC^−/−^ mice. The translocation of NF-κB p65 into the nucleus was increased in lung nuclear extracts from septic WT mice (Fig. 7b). In HDC^−/−^ mice, the nuclear translocation of NF-κB p65 was weak. In line with this finding, nuclear staining for NF-κB p65 was more detectable in WT than in HDC^−/−^ mice after sepsis induction (Fig. 7c).

### Sepsis-induced inflammation and organ injury are alleviated in H_1_-/H_2_-receptor double knockout mice

**Fig. 8:**
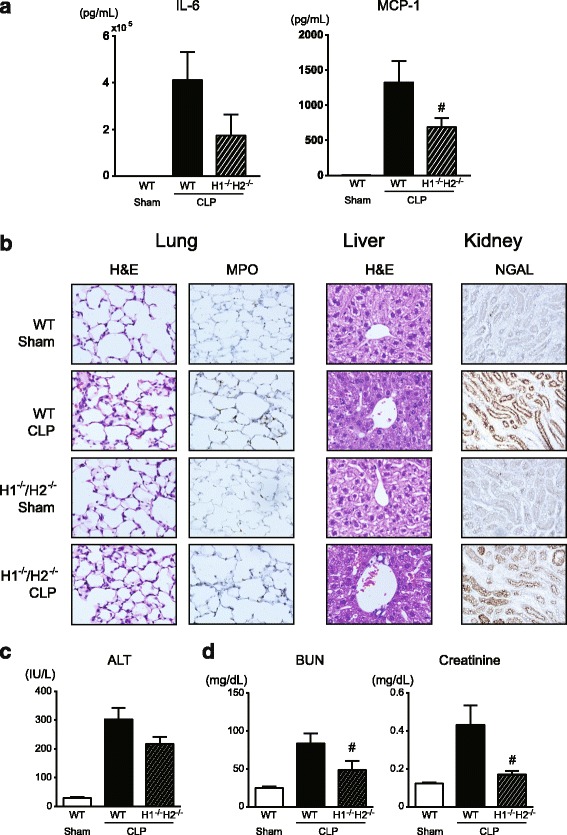
Reduced tissue injury in H_1_R^−/−^/H_2_R^−/−^ mice following CLP-induced sepsis. **a** Blood levels of IL-6 and MCP-1. **b** Representative micrographs of tissue sections stained with hematoxylin and eosin, anti-MPO antibody, and anti-NGAL antibody. Lung, liver, and kidney tissues were harvested from sham-operated and CLP-induced septic mice 24 h after surgery. Original magnification, ×200 or ×400. The same results were obtained with two other experiments. **c** Serum levels of ALT. **d** Serum levels of BUN and creatinine. Blood and tissue samples were taken at 18 h after surgery (*n* = 5–8). All values are provided as means ± SEM. ^#^ *p* < 0.05 vs. CLP WT

When H_1_R^−/−^/H_2_R^−/−^ mice were rendered septic by CLP, the rise in blood levels of cytokines, IL-6 and MCP-1, was evidently attenuated in comparison with WT (Fig. 8a). In addition, H_1_R^−/−^/H_2_R^−/−^ mice exhibited lower levels of IL-1β, IL-6, and MCP-1 mRNAs in lung, liver, and kidney tissues as compared with WT following sepsis (Additional file 2: Figure S2). The histological derangements of the lungs, liver, and kidney following CLP-induced sepsis were reduced in H_1_R^−/−^/H_2_R^−/−^ mice (Fig. 8b). When serum ALT in H_1_R^−/−^/H_2_R^−/−^ mice was measured as a marker indicative of liver damage, the markedly increased level after sepsis was subsided (Fig. 8c). Also, the high levels of serum BUN and creatinine, routine measures of kidney function, observed after sepsis were alleviated in H_1_R^−/−^/H_2_R^−/−^ mice (Fig. 8d).

### Effects of H_1_- and H_2_-receptor antagonists on sepsis-induced inflammation and organ injury

**Fig. 9:**
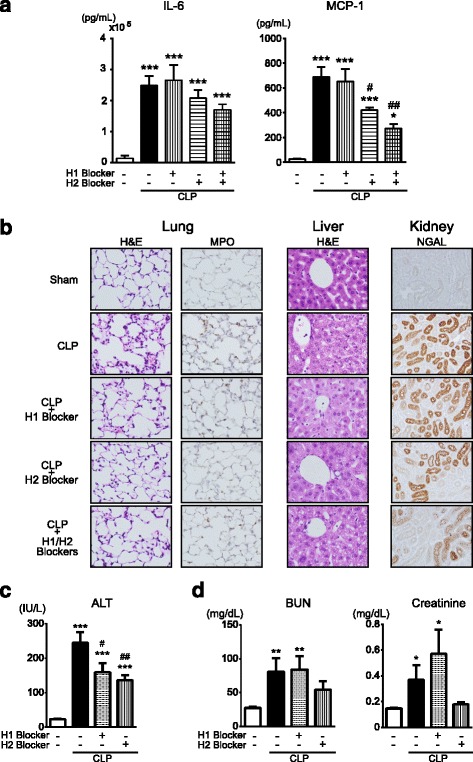
Effects of treatment with *d*-chlorpheniramine and famotidine on elevated blood cytokines and organ injury following CLP-induced sepsis. **a** Blood levels of IL-6 and MCP-1. **b** Representative micrographs of tissue sections stained with hematoxylin and eosin, anti-MPO antibody, and anti-NGAL antibody. Lung, liver, and kidney tissues were harvested from sham-operated and CLP-induced septic mice 24 h after surgery. Original magnification, ×200 or ×400. The same results were obtained with two other experiments. **c** Serum levels of ALT. **d** Serum levels of BUN and creatinine. Blood and tissue samples were taken at 18 h after surgery (*n* = 6–10/group). All values are provided as means ± SEM. *p < 0.05, **p < 0.01, ***p < 0.001 vs. the respective sham control. ^#^ *p* < 0.05, ^##^ *p* < 0.01 vs. CLP alone

Mice were injected intraperitoneally with a single dose of *d*-chlorpheniramine (10 mg/kg) and famotidine (20 mg/kg) 60 min before CLP to block H_1_- and H_2_-receptors, respectively. The elevated blood levels of IL-6 and MCP-1 after sepsis appeared to be reduced more by combined treatment with *d*-chlorpheniramine and famotidine than with famotidine alone (Fig. 9a). Furthermore, the sepsis-induced increases in tissue levels of IL-1β, IL-6, and TNF-α mRNAs were lowered when the two blockers were given together to the animals (Additional file 3: Figure S3).

When CLP-induced septic mice received treatment with *d*-chlorpheniramine, famotidine, or both, the histological damage in the lungs was apparently minimized and the increase in MPO-positive cells was blunted by each treatment (Fig. 9b). In liver histology, *d*-chlorpheniramine, famotidine, or both showed a protective effect on liver damage caused by CLP-induced sepsis (Fig. 9b). The serum ALT levels, which were markedly increased at 18 h after sepsis induction, were reduced by treatment with each of *d*-chlorpheniramine and famotidine (Fig. 9c). In the kidneys, intense accumulation of immunoreactive NGAL that came along with sepsis remained unchanged with *d*-chlorpheniramine, but was attenuated by famotidine alone or combined with *d*-chlorpheniramine (Fig. 9b). Serum BUN and creatinine levels showed no difference between septic mice untreated and treated with *d*-chlorpheniramine, but famotidine treatment blunted the rise in the serum markers (Fig. 9c).

## Discussion

The specific pathophysiology and molecular basis of sepsis-associated multiple organ failure is still not fully understood. The updated definition of sepsis and septic shock has unveiled that organ dysfunction/failure is critical in determining the clinical outcome of sepsis [3]. Here, we provide clear evidence that histamine is identified as an aggravating mediator to contribute to the development of major end-organ (that is, lung, liver, and kidney) injury in sepsis.

Circulating levels of histamine were significantly elevated in mice after induction of polymicrobial sepsis by CLP, as fully demonstrated in our previous report [10]. This elevation in the circulating histamine levels was associated with increased tissue expression of HDC, an enzyme that only forms histamine in mammals. This could result in locally elevated levels of histamine concentrations in tissues. Indeed, we found that histamine levels elevated in the lung, liver, and kidney tissues in a time-dependent manner. In addition, the upregulation of gene expression levels of H_1_- and H_2_-receptors was observed after sepsis induction but quite varied between tissues. Taken together, these data shadow a possible role of histamine in the pathophysiology of sepsis.

Our CLP murine model of sepsis developed lung, liver, and kidney injury, as evidenced by histological changes, neutrophil filtration index, and biochemical variables. We found that sepsis-induced multiple organ injury was significantly attenuated in HDC^−/−^ mice. This suggests that the lack of endogenous histamine could help to reduce sepsis-induced multiple organ injury. Alternatively, we interpret this finding to assume that histamine acts as a mediator to promote the development of multiple organ injury in sepsis. The attenuation of septic organ injury in HDC^−/−^ mice may be partly the result of a reduction in cytokine production. Sepsis triggers overproduction of a diverse set of proinflammatory and chemotactic cytokines as demonstrated in this study. Their uncontrolled, exuberant production can be deleterious to various tissues and can lead to organ injury and dysfunction [24], although the pathogenesis of multiple organ dysfunction is multifactorial and is still being explored [25]. In agreement with the present results on cytokines in HDC^−/−^ mice, LPS-stimulated IL-6 production in liver tissues has been shown to fall to a low level in HDC^−/−^ mice [26]. Moreover, in vitro experiments have reported that histamine increases IL-6 production in B cells and glial cells [27], endothelial cells [28], and peripheral blood mononuclear cells [29], although there is found to be a report showing that histamine suppresses LPS-induced gene expression and synthesis of TNF-α in peripheral blood mononuclear cells mediated by H_2_-receptors [30].

However, our experiments with the H_1_-receptor antagonist *d*-chlorpheniramine and the H_2_-receptor antagonist famotidine indicate that the lessening of sepsis-induced organ injury observed in HDC^−/−^ mice cannot be solely attributed to alterations in proinflammatory and chemotactic cytokine production. These antagonists were not necessarily effective in reducing some cytokines in blood, such as IL-1β and IL-6, which is inconsistent with their changes obtained in HDC^−/−^ mice. Yet, both *d*-chlorpheniramine and famotidine were effective in reducing septic lung and liver injury, whereas famotidine, but not *d*-chlorpheniramine, mitigated septic kidney injury. This suggests that, while both H_1_- and H_2_-receptors are involved in lung and liver injury, only H_2_-receptors contribute to kidney injury in sepsis. The involvement of histamine via H_1_-receptors in lung vascular hyperpermeability in sepsis has been documented [9, 31]. H_2_-receptors have also been shown to be involved in the recruitment of neutrophils and protein leaks in LPS-induced acute lung injury [32]. These adverse effects of histamine mediated by H_1_- and H_2_-receptors could be responsible for liver injury in sepsis. In renal ischemia/reperfusion injury, the beneficial effects of the H_2_-receptor antagonist ranitidine have been found to be partly mediated by decreased IL-6 production [33]. Furthermore, it has been reported that mast cell-deficient mice exhibit attenuated acute kidney injury with cisplatin which is associated with reduced serum TNF-α levels and reduced recruitment of leukocytes to the inflamed kidney [34].

The transcription factor NF-κB has been well recognized as a pivotal player in the pathophysiology of sepsis [35]. NF-κB is involved in regulating the transcription of many of the immunomodulatory mediators that can participate in the development of sepsis-induced organ failure [36]. In a myriad of stimuli, commencing with endotoxin, IκBα is quickly phosphorylated, ubiquitinated, and degraded, releasing the NF-κB heterodimer, which then translocates from cytoplasm into nucleus to mediate the transcription of inflammatory genes. Interestingly, IκBα phosphorylation and degradation following CLP were impaired in lungs of HDC^−/−^ mice. As a result, HDC^−/−^ mice displayed low nuclear levels of NF-κB p65 in CLP-induced sepsis. We interpret these results to indicate that histamine can exert a facilitatory effect on activation of the NF-κB signaling pathway. Thus, histamine may help to promote the development of major end-organ injury in sepsis by enhancing NF-κB activity.

Contrary to the present findings indicative the role of H_2_-receptor activation in worsening septic liver injury, a previous report has demonstrated that histamine pretreatment can ameliorate D-galactosamine/LPS-induced liver injury in WT and H_1_-receptor knockout mice, but not H_2_-receptor knockout mice [37]. Furthermore, histamine through H_2_-receptors has been documented to protect the liver against alcohol-induced injury in rats [38]. It is difficult to reconcile these findings at present, but possible reasons for the apparent discrepancy may include different regulatory mechanisms between systemic vs. local inflammation and concentration-related differences between endogenous vs. exogenous histamine.

It is now well established that histamine exerts its biological effects by binding to and activating four distinct separate receptors: H_1_-, H_2_-, H_3_-, and H_4_-receptors [12]. Although our experiments with *d*-chlorpheniramine and famotidine imply that H_1_- and H_2_-receptors are involved in the development of septic organ injury, we cannot entirely exclude that the lack of activation of H_3_- and H_4_-receptors may contribute to reduced organ injury in HDC^−/−^ mice following sepsis. Interestingly, H_4_-receptors appear to play a role in sepsis-associated induction of apoptosis in the key organs [10]. The exact role of H_3_- and H_4_-receptors in the pathophysiology of sepsis awaits further study using the animals deleted for their genes. It should be noted, however, that H_1_R^−/−^/H_2_R^−/−^ mice displayed lesser degree of sepsis-related organ injuries as seen in HDC^−/−^ mice.

Clinically, H_1_-receptor antagonists may be prescribed in perioperative settings, since many narcotics can induce itching [39]. H_2_-receptor antagonists are widely used in critically ill patients to reduce the risk of gastrointestinal bleeding [40, 41]. Intriguingly, a significant risk for hospital-acquired pneumonia has been found for proton pump inhibitor (PPI) use but not H_2_-receptor antagonists in hospitalized patients [42], although the findings of an updated meta-analysis to evaluate the effects of PPIs vs. H_2_-receptor antagonists on clinically gastrointestinal bleeding in critically ill patients have shown no differences between drugs in the risk of pneumonia, death, or ICU length of stay [43]. Whether H_2_-receptor antagonists vs. PPIs, when used for the prevention of gastrointestinal bleeding in the ICU, can differently affect the development of sepsis-associated organ failure awaits future clinical trials.

## Conclusions

This study sheds light on the new role of histamine in the pathophysiology of sepsis. We represent the first report that endogenous histamine acting on H_1_- and H_2_-receptors is identified as an aggravating mediator to contribute to the development of major end-organ (that is, lung, liver, and kidney) injury in sepsis. While our present study suggests the benefit of their treatment in reducing sepsis disorder and supports that they may be safe medications in critically ill patients with sepsis, the validity and feasibility of the use of these histamine receptor antagonists to avoid the development of septic organ injury warrant further clinical investigations and evaluation.

## Additional files

Additional file 1: Figure S1. Changes in plasma concentrations of histamine in mice after CLP. A hydrophilic interaction liquid chromatography-mass spectrometry method was used for the quantitative determination of histamine. **p* < 0.05 vs. time 0; *n* = 4/group. (PNG 4 kb)
Additional file 2: Figure S2. Transcription levels of IL-1β, IL-6, and MCP-1 in lung, liver, and kidney tissues of H_1_R^−/−^/H_2_R^−/−^ mice following CLP-induced sepsis. Tissues were harvested at 18 h after surgery (*n* = 5–8/group). The values were expressed as a fold increase above sham-operated WT normalized GAPDH. (PNG 10 kb)
Additional file 3: Figure S3. Transcription levels of IL-1β, IL-6, and TNF-α in lung, liver, and kidney tissues of *d*-chlorpheniramine- and famotidine-treated mice following CLP-induced sepsis. Tissues were harvested 18 h after surgery (*n* = 7–15/group). The values were expressed as a fold increase above sham-operated control normalized GAPDH. (PNG 8 kb)
