# Supplementary figures and images for: Critical role of endogenous histamine in promoting end-organ tissue injury in sepsis
Source: Intensive Care Med Exp. 2016 Nov 8;4:36. doi: 10.1186/s40635-016-0109-y (PMC5099302; doi:10.1186/s40635-016-0109-y)

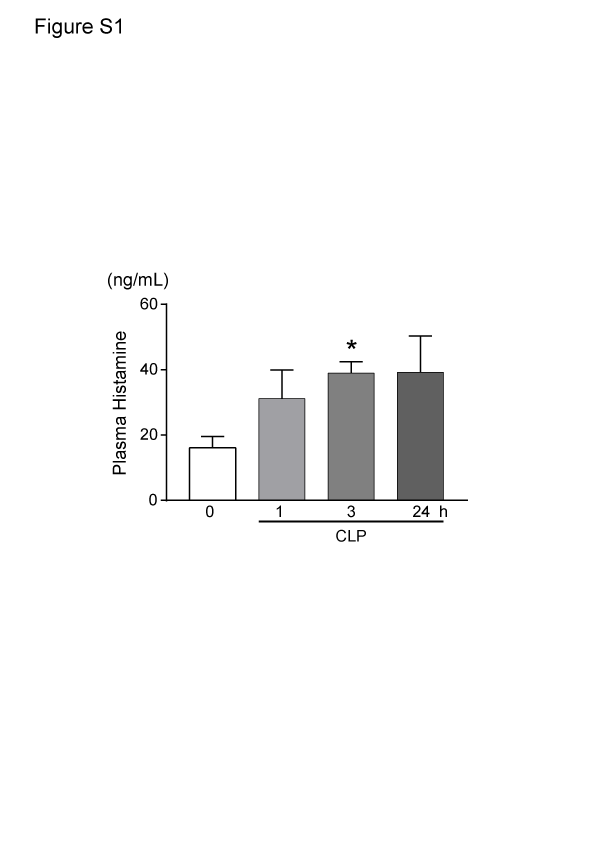

Supplement: Additional file 1: Figure S1. — Changes in plasma concentrations of histamine in mice after CLP. A hydrophilic interaction liquid chromatography-mass spectrometry method was used for the quantitative determination of histamine. *p < 0.05 vs. time 0; n = 4/group. (PNG 4 kb) [file 40635_2016_109_MOESM1_ESM.png]

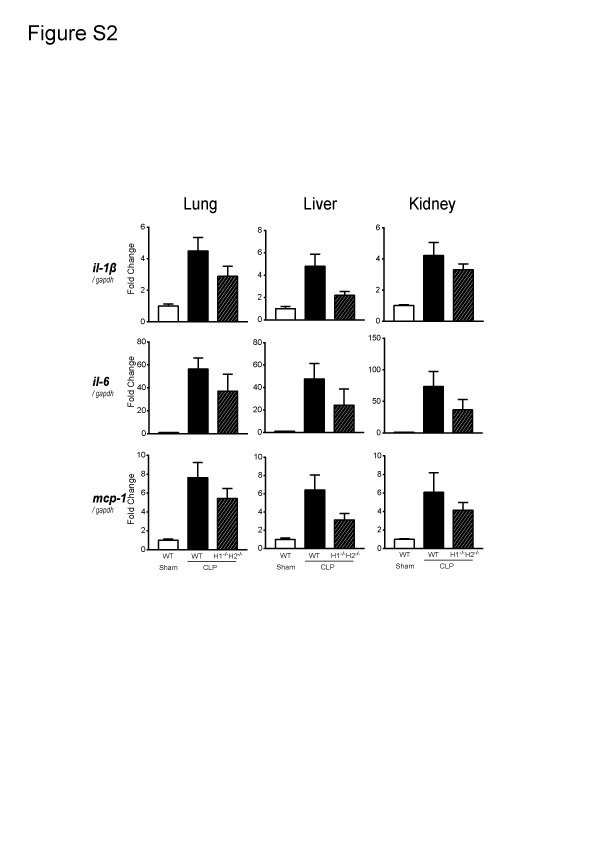

Supplement: Additional file 2: Figure S2. — Transcription levels of IL-1β, IL-6, and MCP-1 in lung, liver, and kidney tissues of H1R−/−/H2R−/− mice following CLP-induced sepsis. Tissues were harvested at 18 h after surgery (n = 5–8/group). The values were expressed as a fold increase above sham-operated WT normalized GAPDH. (PNG 10 kb) [file 40635_2016_109_MOESM2_ESM.png]

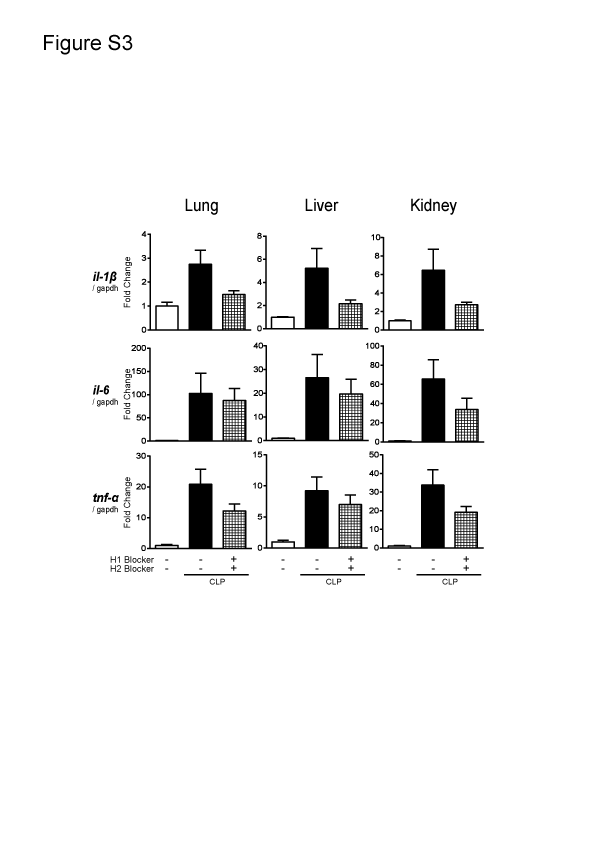

Supplement: Additional file 3: Figure S3. — Transcription levels of IL-1β, IL-6, and TNF-α in lung, liver, and kidney tissues of d-chlorpheniramine- and famotidine-treated mice following CLP-induced sepsis. Tissues were harvested 18 h after surgery (n = 7–15/group). The values were expressed as a fold increase above sham-operated control normalized GAPDH. (PNG 8 kb) [file 40635_2016_109_MOESM3_ESM.png]
